# Survey-Based Evaluation of the Impact and Effectiveness of a Nuclear Medicine Training Program: An Insight from Graduate Trainees

**DOI:** 10.7759/cureus.90479

**Published:** 2025-08-19

**Authors:** Veronika Svistkova, Bilquis Hyder Ali, Eugene Leung, Alireza Khatami

**Affiliations:** 1 Division of Nuclear Medicine and Molecular Imaging, Department of Medicine, The Ottawa Hospital, University of Ottawa, Ottawa, CAN

**Keywords:** fellow education, medical education, nuclear medicine, nuclear medicine imaging, residency program

## Abstract

Background: Residency and fellowship training in nuclear medicine aims to equip specialists with diagnostic, technical, clinical, and research skills essential for clinical and academic roles. This study evaluates the effectiveness of a nuclear medicine training program as perceived by former trainees, focusing on mentorship, resources, curriculum content, and professional development.

Objective: The objective of the study was to evaluate the perceived impact of nuclear medicine training programs on clinical competence, professional development, and career readiness, based on feedback from former trainees.

Methods: In 2024, an online survey was administered to former trainees of the nuclear medicine training program of the University of Ottawa, Canada, across multiple countries, including Canada, Saudi Arabia, Iran, and Oman. Responses from the 11 participants assessed satisfaction with mentorship, facilities, off-service rotations, and skill application post-training. Statistical analysis, including analysis of variance (ANOVA), evaluated the program's impact.

Results: The program received high ratings for overall quality (45% (n=5) excellent, 35% (n=4) very good) and its contribution to professional success (85% (n=9) significantly or very significantly). The program was rated very effective by 65% (n=7) of respondents. Key strengths included hands-on training, access to technology, and diagnostic skill development. Areas for improvement included mentorship consistency, advanced equipment access, and structured leadership training.

Conclusions: The nuclear medicine residency and fellowship program effectively enhances diagnostic and clinical competencies. Enhancing mentorship, technology access, leadership development, and research support will optimize training outcomes.

## Introduction

Postgraduate training in nuclear medicine is essential for preparing specialists in an increasingly interdisciplinary field that encompasses hybrid imaging, molecular diagnostics, and theranostics. As clinical complexity increases, training programs must balance technical expertise with leadership, scholarship, and communication skills [1].

Despite this evolution, few nuclear medicine residency programs have been systematically evaluated from the perspective of those who experience them: the trainees. Understanding their motivations, satisfaction, and barriers is essential for workforce planning and curricular reform. While nuclear medicine specialty training offers many advantages, it also has some uncertainties. A large national survey of residents in an integrated nuclear medicine and radiology residency program in the Netherlands, for example, found that fewer trainees are selecting nuclear medicine as a subspecialty than anticipated, only 14 out of 175 eligible residents at the time of the study in 2021 [2]. While key attractions included access to theranostics, strong academic environments, and hybrid imaging, disincentives included a perceived imbalance in training time, uncertainty about career prospects, and limited early exposure to nuclear medicine during medical school [2]. Understanding these challenges is essential to improving training programs and attracting future specialists.

Starting as early as medical school, mentorship is considered a valuable tool that supports medical students from early in their training, with demonstrated benefits such as increased research productivity and improved residency match success. However, several barriers limit the effectiveness and availability of mentorship programs, including faculty time constraints, inadequate mentor training, and the limited availability of BIPOC (Black, Indigenous, and People of Color) and female mentors [3].

In nuclear medicine, mentorship plays a critical role in shaping trainees' clinical and academic competencies and is one of the most effective strategies for strengthening training outcomes. It facilitates the development of clinical, academic, and personal competencies through structured, longitudinal guidance. Effective mentorship is not about providing all the answers but about fostering independent growth, helping trainees identify their goals, and guiding them along a purposeful path. In nuclear medicine, where training needs can vary widely, from research-focused academic careers to private practice, mentorship helps personalize the educational journey and enhance professional readiness [4].

Structured mentorship models, whether one-on-one or group-based, rely on clarity, feedback, and mutual engagement. As the specialty grows in scope and complexity, flexible mentorship programs can support diverse learning needs across different career stages. Recognizing this, professional societies such as the European Association of Nuclear Medicine (EANM) have implemented formal mentorship initiatives to build continuity between trainees and senior faculty and to cultivate future leaders in the field [5].

Other ways to improve resident satisfaction include the use of interactive learning strategies. A recent study from Sichuan Provincial People’s Hospital, conducted among first-year nuclear medicine residents, demonstrated that combining Small Private Online Courses (SPOCs) with a flipped classroom approach significantly improved resident satisfaction, professional competence, patient care, and learning capabilities. In this model, residents engaged with short pre-recorded lectures and quizzes before class, then participated in case-based discussions, peer presentations, and group problem-solving during class time. These results highlight how active, learner-centered formats can foster deeper understanding and better clinical readiness in nuclear medicine training compared to traditional lecture-based methods [6].

Considering these insights, we were inspired to perform our study and evaluate our program for potential changes in the future. Our study aims to evaluate the nuclear medicine residency and fellowship program of the University of Ottawa, Ottawa, Canada, through a survey of former trainees. The training program’s goal is to prepare specialists in nuclear medicine who are equipped with diagnostic, technical, clinical, and research skills necessary for both clinical and academic roles. The evaluation was done through a questionnaire designed to assess various dimensions of the training, including quality of mentorship, communication, available resources, curriculum content, and post-training professional success. Participants provided feedback on the training's impact on their clinical competence, research opportunities, and overall career advancement. The primary objective of this exploratory study was to provide a comprehensive assessment of the program from the perspective of former trainees; no secondary aims were planned.

## Materials and methods

This study employed a cross-sectional survey design to evaluate the perceived impact and effectiveness of a nuclear medicine residency and fellowship training program. An anonymous online survey was distributed to former trainees of the Nuclear Medicine Program of the University of Ottawa, who had completed their training between 2010 and 2023. Participants were recruited through alumni records and professional networks, and were invited via email to participate voluntarily. Twelve participants received the questionnaire; 11 completed it, while one former trainee was unreachable despite multiple follow-up attempts. Ethical approval was sought from the Ottawa Health Science Network Research Ethics Board, and was issued exempt.

The questionnaire was developed using Microsoft Forms (Microsoft Corporation, Redmond, Washington, United States) and comprised 14 items, including multiple-choice, Likert-scale, and ranking questions. The survey captured demographic information (for example, country of training, current professional role, time since graduation), areas of clinical specialization, and subjective evaluations of the training program. Specific domains assessed included the perceived quality of mentorship, access to resources, structure of the curriculum, effectiveness of off-service rotations, and impact on professional development. Respondents also rated how the training influenced various competencies such as diagnostic skills, clinical decision-making, interdisciplinary collaboration, and leadership.

Descriptive statistics (frequencies, percentages) were used to summarize categorical responses, while rankings were aggregated to highlight the most valued aspects of the training. We additionally report results from single-factor ANOVA analyses conducted on the original survey scales to identify possible differences in trainee-reported outcomes. Given the small sample size (n = 11), these inferential results are presented as exploratory only. Frequencies and percentages were calculated for categorical responses, and rankings were aggregated to identify the most and least valued aspects of the training experience. Open-text responses were reviewed to identify common themes related to strengths and areas for improvement in the program.

## Results

Demographics of participants

Participants in this study were graduates from Canada (n=5, 45%), Saudi Arabia (n=4, 36%), Iran (n=1, 9%), and Oman (n=1, 9%). They held various positions in clinical and research fields, including practicing as attending staff (n=11, 100%), of which three were academic faculty (27%) and three were researchers (27%). Eight (73%) participants had dual certificates in radiology and nuclear medicine, eight (73%) reported general nuclear medicine studies, seven (63.65%) were involved with nuclear cardiology, six (55%) were involved with positron emission tomography-computed tomography (PET-CT) reporting, and two (18%) were involved in radioligand therapy. Among the respondents, five (45%) had more than five years’ experience in their current job, and six (55%) had less than five years of working experience.

Overall program evaluation, effectiveness, and trainees’ satisfaction

**Figure 1:**
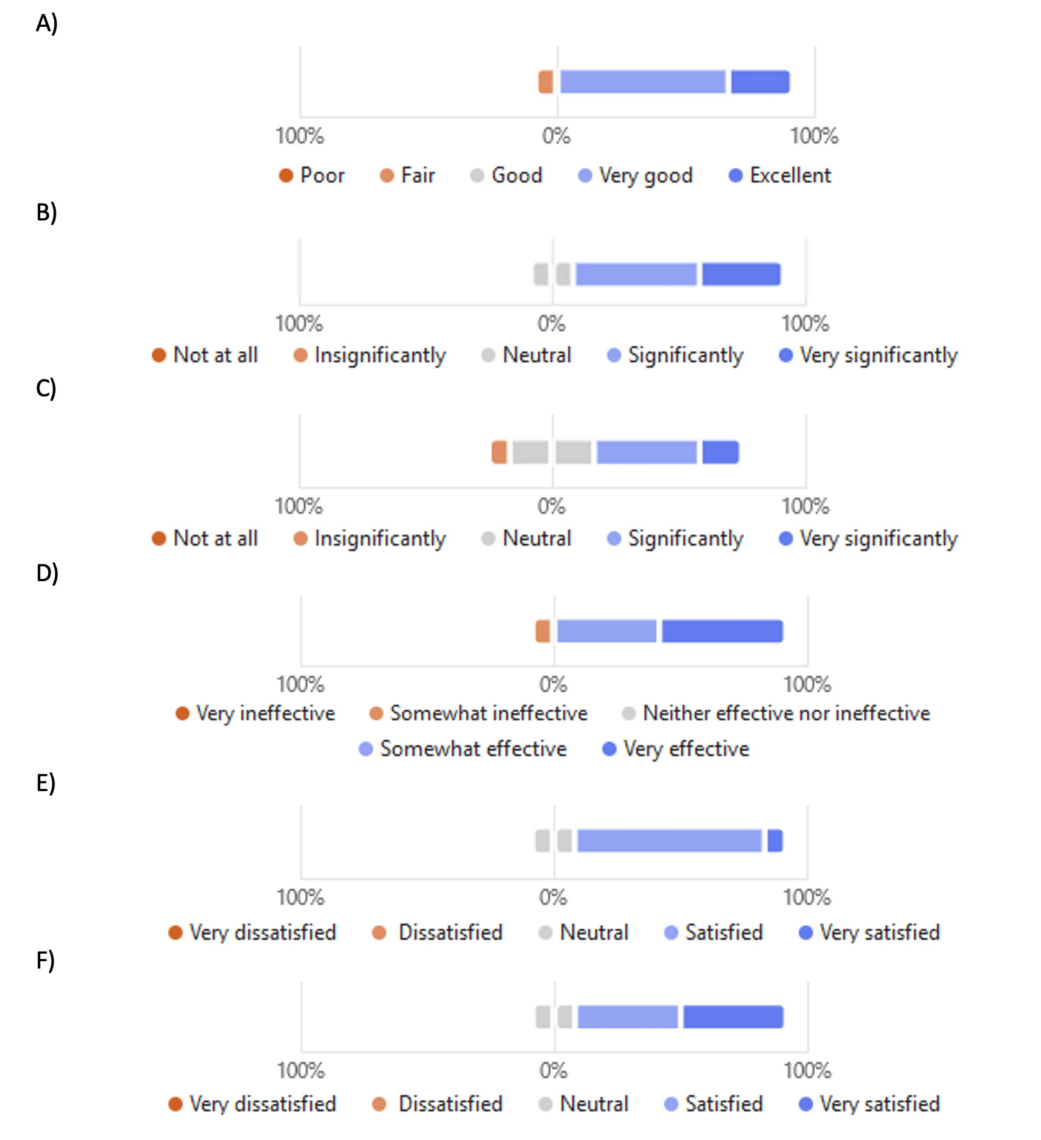
Program quality, significance, effectiveness and satisfaction A) overall quality of the training program at university of Ottawa, B) the extent of training program contribution to professional success of trainees, C) the significance contribution of training program to the advancement of nuclear medicine in the region of trainees practice, D) Effectiveness of the mentorship provided during your training, E) trainees’ satisfaction regarding the resources, and F) off-service rotations provided during training.

Overall, the quality of the training program was rated excellent by three (27%) and very good by seven (64%). Only one (9%) out of the 11 participants rated the quality as fair (Figure 1A). The training program contributed to the professional success of trainees very significantly in three (27%) or significantly in six (55%) participants, while two (18%) remained neutral in this regard with no negative rating (Figure 1B). The training program contributed to the advancement of nuclear medicine in the region where the trainee is practicing very significantly for two (18%) and significantly for four (36%) participants, while four (36%) remained neutral. Only one (18%) rated that the program insignificantly contributed to the advancement of nuclear medicine regionally (Figure 1C). The trainees received mentorship as part of the training program, and they rated the mentorship overwhelmingly positive; it was rated as very effective by five (45.5%) and somewhat effective by five (45.5%) participants while the remaining one (9%) participant rated it as somewhat ineffective (Figure 1D). Regarding trainees’ satisfaction, one (9%) was very satisfied, eight (73%) were satisfied, while two (18%) were neutral in this regard (Figure 1E). With regard to off-service rotation offered by the training program, five (45.4%) were very satisfied, four (36.4%) were satisfied, and two (18.2%) remained neutral (Figure 1F).

**Figure 2:**
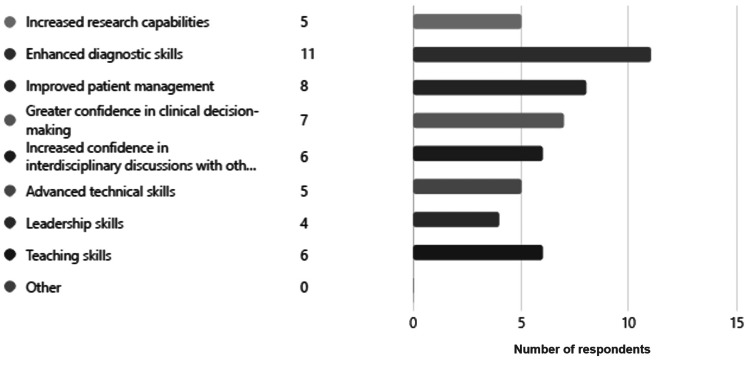
Field application of the knowledge and skills gained from the training program in the region the graduate from the training program practiced in.

**Figure 3:**
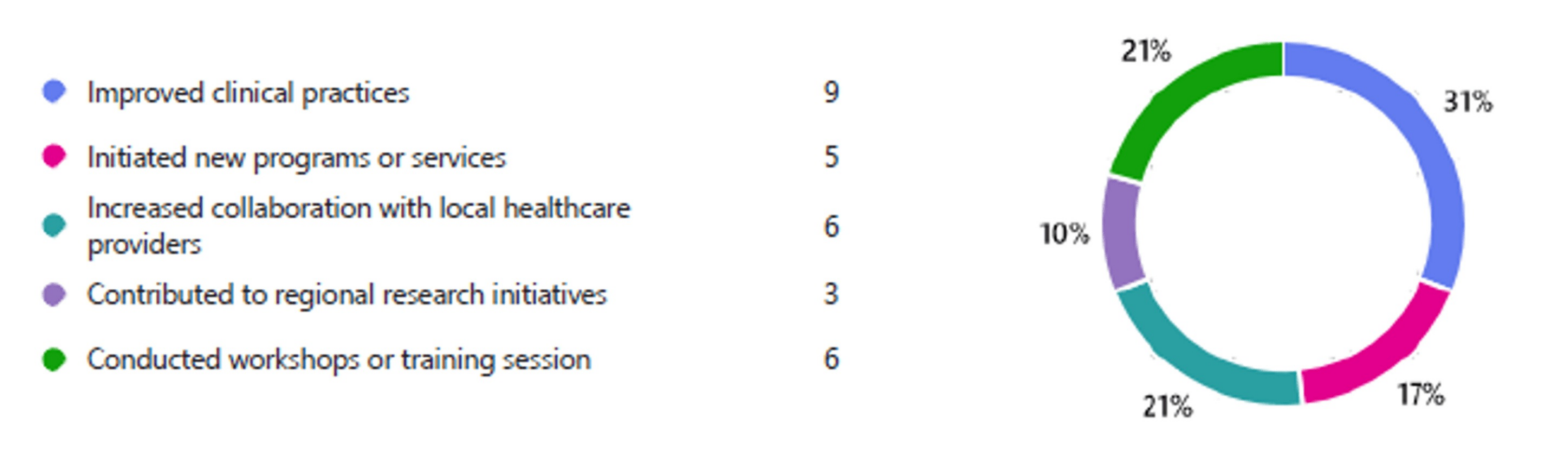
Application of the knowledge and skills gained from the training in region of practice.

The training program helped trainees with knowledge and skills for their future practice by enhancing their diagnostic skills, improving patient management, improving confidence in clinical decision-making, increasing confidence in interdisciplinary discussion with other specialties, and teaching skills (Figure 2). The experience that was gained during the training program mostly helped the trainees improve clinical practice, increase collaboration with other local healthcare providers, and conduct workshops or training sessions, as well as initiate new programs or services in the department they are practicing (Figure 3). 

Assessment of the training program based on trainee feedback

For a detailed evaluation of the training program, a series of questions was designed and structured into three categories: the most beneficial aspects of the program, areas for improvement, and suggested additional resources or supports. Each category provided insight into trainees' priorities, concerns, and suggestions for enhancement. The trainees were asked to rank the question items based on priority and importance. The following are the rank-wise results.

The Most Beneficial Aspects of the Training Program

Hands-on clinical training: Hands-on clinical training ranked highest, indicating that trainees highly valued direct experience with diverse patient cases and advanced imaging techniques. This emphasizes the importance of experiential learning in reinforcing knowledge and enhancing clinical skills.

Mentorship and support: Mentorship, ranked second, highlighting the importance of faculty guidance and regular feedback. Mentorship was rotation‑based, with a supervising faculty member assigned to each block. Trainees requiring additional support, remediation, or focused skill development were assigned to a dedicated one‑on‑one mentor. Trainees appreciated personalized support for their professional development, which seems integral to their growth and confidence in clinical settings. 

Guest lectures: City-wide grand rounds and case rounds with guest experts were highly valued. Exposure to diverse perspectives from invited lecturers enriched the learning experience and broadened trainees' clinical insights beyond the program's immediate scope.

Curriculum structure: A well-organized curriculum balancing theory and practice was appreciated, suggesting that trainees valued a structured program that helps them efficiently absorb and apply knowledge.

Learning environment: A positive and inclusive learning environment is important, as it fosters supportive peer relationships and collaborative learning, which enhanced the overall training experience.

Research opportunities: Although research opportunities were valued, they ranked lower than hands-on training and mentorship. Trainees saw research as beneficial but secondary to the practical and mentorship-driven aspects of their education.

Access to technology: Modern equipment and new tool training were appreciated but ranked lower. This suggests that while technology played a role in clinical skills development, it was not as central as hands-on experience and mentorship.

Networking and collaboration: Networking opportunities were seen as valuable but less prioritized compared to clinical training and mentorship, indicating that building professional connections was not as crucial during training.

Work-life balance: Although work-life balance was important, it ranked lower, suggesting that clinical learning and mentorship were prioritized over flexibility in work hours or scheduling.

Professional development: Workshops and career advancement support ranked the lowest, indicating that while helpful, professional development was seen as peripheral compared to other core training elements.

Areas That Could Be Improved

Facilities and resources: The need for upgraded facilities, equipment, and better access to learning materials was the highest-ranked area for improvement. This indicated that better infrastructure was critical to supporting the program’s practical and educational components.

Evaluation and feedback: Trainees requested more frequent evaluations, clearer assessment criteria, and peer feedback. A more structured and transparent feedback system could help trainees identify strengths and areas for improvement.

Communication: There was a call for improved communication, particularly in feedback and interdepartmental interaction. Better communication would ensure smoother operations and a better flow of essential information.

Curriculum content: Respondents wanted updates to the curriculum, with new topics and better integration of theory and practice. This suggests a need for the program to evolve and stay up-to-date with medical advancements and address gaps between theory and practice.

Clinical training: While clinical training was highly regarded, there was a request for a greater variety of cases and more hands-on experiences. Expanding clinical exposure would better prepare trainees for diverse real-world scenarios.

Mentorship: There was a demand for increased mentor availability and regular check-ins. Although mentorship was highly valued, improving its accessibility could further support trainees’ development.

Didactic lectures: More grand rounds and case reviews were requested, signaling that trainees would benefit from more opportunities to learn from expert-led discussions on complex cases.

Networking opportunities: Respondents sought more collaboration opportunities with other institutions, suggesting that existing networking events may not be sufficient in fostering professional connections beyond the program.

Research opportunities: Enhanced support for independent research projects and increased collaboration with research teams were desired. This reflected a desire for deeper engagement with research and academic contributions.

Suggested Additional Resources or Supports

More guest lecturers: There was a strong interest in additional expert talks and webinars on current practices. Respondents valued external insights that helped them stay up-to-date and broaden their clinical perspectives.

More case review sessions: More opportunities for case discussions, particularly challenging cases, were requested. These sessions foster collaborative learning and help trainees refine their problem-solving skills in a supportive environment.

Peer study groups: Collaborative study sessions among fellows were seen as valuable. Peer interactions were important for reinforcing concepts, sharing insights, and problem-solving collectively.

Feedback box: An anonymous suggestion box for program improvements would provide a safe space for trainees to voice concerns or suggest changes. This would enhance feedback mechanisms and program transparency. 

Table 1 summarizes the above-mentioned findings.

**Table 1:** The rank order list of (A) the most beneficial aspects of training program, (B) areas that could be improved, and (C) suggested additional resources or support for improving training experience.

Parameters	Rank order list
(A) The most beneficial aspect of training program	
Hands-on clinical training; Practical experience with diverse patient cases and advanced imaging techniques	1
Mentorship and support; Guidance from faculty and regular feedback on performance	2
Guest lectures; City-wide grand rounds and Interesting case rounds; Invited experts to give talks	3
Curriculum structure; Well-organized schedule covering topics with a balance between theory and practice	4
Learning environment; Positive and inclusive atmosphere with supportive peer relationships	5
Research opportunities; Participation in ongoing studies and opportunities to present findings	6
Access to technology; Availability of state-of-the-art equipment and training on new tools and software	7
Networking and collaboration; Building professional connections through collaborative projects and meetings	8
Work-life balance; Reasonable work hours along with flexibility in scheduling	9
Professional development; Access to workshops and support for career advancement	10
(B) Areas that could be improved	
Facilities and resources; Upgraded equipment and technology, improved access to learning materials	1
Evaluation and feedback; More frequent evaluations, clearer assessment criteria, and opportunities for peer feedback	2
Communication; Improved feedback mechanisms and better intradepartmental communication	3
Curriculum content; New topics, in-depth coverage of key areas, and better integration of theory and practice	4
Clinical training; More hands-on experiences and a greater variety of cases	5
Mentorship; Increased mentor availability and regular check-ins	6
Didactic lectures; More city-wide grand rounds and interesting case rounds	7
Networking opportunities; More networking events and collaboration opportunities with other institutions	8
Research opportunities; Enhanced support for personal research projects, and more collaboration with research teams	9
(C) Suggested additional resources or supports	
More guest lecturers; Expert talks or webinars on current practice	1/ equally ranked
More case review sessions; Discussions of challenging cases for shared learning	1/ equally ranked
Peer study group; Collaborative learning sessions among fellows	2
Feedback box; Anonymous suggestion for program improvements	3

Program component evaluation

Mentorship

While feedback regarding mentorship was generally positive, some respondents expressed the need for more consistent and regular mentorship. Increased availability of mentors, especially for junior consultants and researchers, was recommended.

 Satisfaction with Resources Provided

Satisfaction levels with training resources, such as materials and technology, were positive but indicated a need for enhanced access and upgraded equipment, particularly in advanced technologies such as single photon emission computed tomography (SPECT) and PET scanners.

Off-Service Rotations

Participants noted varied experiences with off-service rotations (e.g., pediatric nuclear medicine, nuclear cardiology, PET-CT), highlighting both the value of these rotations for a comprehensive training experience and areas for improvement in exposure and training quality.

Application of Knowledge and Skills

The training contributed to improved clinical practice, enhanced collaboration, conducting research, and leadership in new program initiatives.

Additional Resources and Support

Respondents identified several areas where additional resources or support could enhance the training experience such as: (i) More case review sessions: Collaborative learning through case reviews was highly valued; (ii) Guest lectures and expert talks*:* Increased exposure to current practices and new developments through expert lectures and webinars was suggested; (iii) Peer study groups:Collaborative sessions among trainees were seen as beneficial, offering opportunities for peer-to-peer learning and mutual support; (iv) Feedback mechanisms:A suggestion was made for implementing an anonymous feedback box to encourage candid suggestions and concerns.

**Figure 4:**
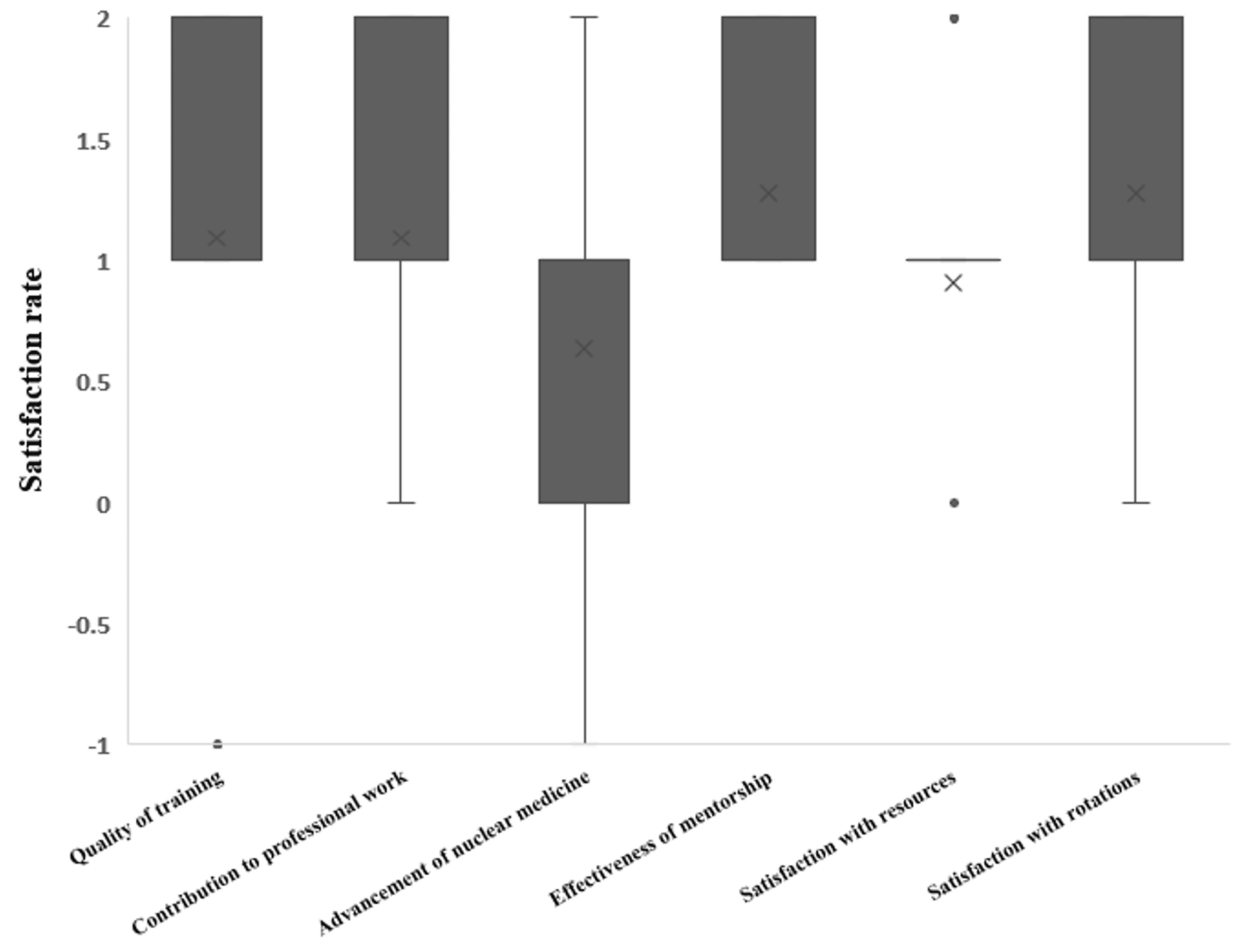
Program effectiveness and satisfaction based on the Likert questionnaire Median and mode of satisfaction/ effectiveness of the training program in a five- scale from rating question (-2= completely not satisfied to 2= extremely satisfied and 0 is Neutral). The single-factor ANOVA indicated associations between participation in the Nuclear Medicine Training Program and several key aspects of professional development. Specifically, the analysis showed effects on diagnostic skills (p = 0.002), confidence in interdisciplinary communication and collaboration (p = 0.009), decision-making capabilities (p = 0.026), and teaching skills (p = 0.009). However, given the limited sample size, these p-values are presented as exploratory signals rather than definitive evidence. We therefore emphasize descriptive patterns and the distribution of ratings and recommend that these findings be confirmed in larger, prospectively powered studies.

A summary of the program effectiveness and satisfaction is presented in Figure 4. 

## Discussion

The program received positive feedback, contributing significantly to trainees' professional development and success. Most respondents reported improvements in critical skills such as diagnostic capabilities, patient management, and clinical decision-making, indicating the program's effectiveness in preparing them for various roles within nuclear medicine.

However, several areas for improvement were identified. Mentorship was a key concern, with trainees requesting more structured, consistent guidance. This aligns with recent findings that effective mentorship is vital for professional development during residency training and beyond, it promotes clinical performance and personal wellness [7]. In faculties where one-to-one mentorship between faculty and trainee is difficult due to the lack of faculty members, faculty-resident/fellow group mentorship is a possible solution to ensure all trainees have access to mentorship [8]. Mentorship programs could also help to identify trainees who are at high-risk of poor academic or clinical performance [9].

Additionally, while facilities and resources were generally sufficient, access to advanced technologies and updated equipment could be improved. The program's curriculum also showed room for enhancement, with participants suggesting the inclusion of more advanced topics and greater integration between didactic learning and hands-on training. Recent literature emphasizes the need to modernize training curricula by incorporating both functional and anatomical imaging skills [10]. In addition to remaining relevant, the curriculum should incorporate emerging technologies like hybrid imaging and AI-assisted diagnostics. For example, incorporating hybrid imaging techniques (SPECT/CT, PET/CT, PET/MRI), integrating emerging technologies such as artificial intelligence and deep learning-based image synthesis, which are rapidly shaping clinical practice in nuclear medicine, would be important for future curriculum developments [11]. These innovations not only improve image quality and diagnostic capabilities but also support safer, low-dose imaging and more personalized care, underscoring the importance of equipping trainees with skills relevant to future-ready nuclear medicine practice. 

Networking opportunities were also highlighted, with many respondents expressing a desire for more professional events and collaboration opportunities to expand their networks. Recent literature underscores that networking is a critical driver of career advancement in academic medicine, granting access to mentorship, research collaborations, visibility, and sponsorship-all of which shape professional trajectories [12]. However, access to these networks is often unequal, and training programs have not systematically prepared residents to recognize or utilize networking effectively. Institutions benefit when trainees are supported in building inclusive and broad networks, which foster innovation and cross-functional collaboration. Respondents in our survey also emphasized the need for greater research support, suggesting that structured networking and collaboration would especially benefit those pursuing academic careers in nuclear medicine.

Interestingly, despite its inclusion in the curriculum, leadership training was rated lower, suggesting the need for more focused development in this area. This indicates that, although leadership training is an important aspect of the program, it may require further emphasis to align with the needs and expectations of participants. Although leadership skills tend to improve with experience and over time, it has been identified that inclusion of individual or group project work and of mentoring produces better organizational outcomes and improves leadership [13]. This aligns with the principles of authentic leadership, a human-centered leadership model shown to enhance collaboration, motivation, and staff satisfaction in nuclear medicine settings. The emphasis on self-awareness, transparency, and purpose-driven leadership may offer a sustainable model for developing the next generation of nuclear medicine leaders [14]. Furthermore, recent perspectives emphasize the need for formal leadership pathways within nuclear medicine training, including integration of innovation, change management, and team-building principles to prepare future leaders for evolving clinical and academic demands [15]. Incorporating leadership courses, workshops, and mentorship can address this need, preparing trainees for administrative and academic roles.

Strengths

The program was recognized for its comprehensive training across key domains of nuclear medicine, including PET-CT, radioligand therapy, and nuclear cardiology, allowing trainees to specialize and take on leadership roles in their respective areas. Graduates reported high levels of satisfaction with the overall quality of the training, citing significant contributions to their professional success. Additionally, the program was noted to have a strong positive impact on the development of diagnostic skills, clinical decision-making, and patient management.

Areas for Improvement

Respondents identified several areas requiring enhancement, including the consistency and availability of mentorship, particularly for junior consultants and research-oriented trainees. There was a clear call for greater emphasis on leadership development, as well as improved access to updated resources, advanced imaging equipment, and diverse learning materials. Suggestions also included enriching the curriculum with more hands-on experiences, expanding opportunities for interprofessional networking, and increasing institutional support for personal research initiatives and collaborative academic work. Table 2 summarizes key recommendations proposed by the respondents to improve the structure and delivery of the training program.

**Table 2:** Recommendations for program improvement AI: artificial intelligence

Recommendation	Description
1. Structured mentorship programs	Develop consistent faculty-led and peer mentorship models to provide sustained guidance and address situations where one-on-one mentoring is limited [8].
2. Leadership training modules	Integrate leadership training into the curriculum through individual and group project work, with a focus on mentoring frameworks and developing confidence in leadership roles [15].
3. Research support	Allocate dedicated funding and institutional resources to promote trainee-led research, interdisciplinary collaboration, and structured research education.
4. Enhanced access to technology	Improve access to advanced imaging modalities and simulation-based tools, particularly for training in hybrid and AI-assisted imaging techniques.
5. Professional development opportunities	Expand opportunities for networking, conference participation, and cross-disciplinary collaboration to support long-term career development.

Limitations

This study has several limitations. The small sample size (n = 11) reduces statistical power and increases the risk of both type I and type II errors; therefore, the statistics reported here, particularly the ANOVA p‑values, are presented as complementary analyses and are not intended as strong confirmatory evidence of effect. The reliance on self‑reported data and the absence of objective performance measures further limit causal inferences. Additionally, as the study relied on self-reported data, there is potential for response bias, including social desirability, recall, and self-selection effects, which may have influenced how participants rated their training experience.

However, the findings remain useful to illustrate the performance and impact of a small training program on trainees’ careers. Future research should validate these preliminary results in larger, prospectively powered cohorts and, where feasible, incorporate objective performance metrics to strengthen causal interpretation. 

## Conclusions

The evaluation of the nuclear medicine training program of the University of Ottawa reveals that, overall, the program is highly effective in preparing residents for successful careers in nuclear medicine. The majority of respondents reported significant gains in both their clinical and research skills, with many noting a positive impact on their professional development. While the program is generally well-regarded, there are areas for improvement, particularly in mentorship, resources, and networking opportunities. Addressing these concerns will help further strengthen the program and better support residents in achieving their career goals.

This study presents a comprehensive evaluation of the program, providing actionable insights that can be used to improve training experiences for future trainees. Further studies with larger participant groups across diverse regions will help validate these findings and guide continued program enhancement.
